# *Being and doing in the outdoors brings something extra!* Evaluating the Danish *Healthy in Nature* Project

**DOI:** 10.1080/17482631.2021.1983947

**Published:** 2021-10-29

**Authors:** Søren Andkjær, Trine Top Klein-Wengel, Astrid Ishøi, Christina Bjørk Petersen

**Affiliations:** a Active Living, Department of Sport Science and Clinical Biomechanics, University of Southern Denmark, Odense M, Denmark; b National Institute of Public Health, University of Southern Denmark, Copenhagen K, Denmark

**Keywords:** Nature, the outdoors, *friluftsliv*, health, well-being, Self-Determination Theory, evaluation

## Abstract

**Purpose:**

Little is known of the potential of using nature and outdoor activities in relation to community-based health promotion programmes. This study seeks a better understanding of how people with mental or chronic physical health problems experience a local outdoor health promotion or rehabilitation programmes and a better understanding of how these programs contribute to the participant’s health and well-being.

**Methods:**

The study is based on data from the Healthy in Nature project targeting adults with chronic physical health problems and adults with mental health problems. Data was collected using a qualitative multiple case study design involving five selected cases with both qualitative interviews and observation. Data was analysed using Braun et al.’s 6-phase guide to qualitative reflexive thematic analysis, employing Self-Determination Theory as a theoretical framework. Results: Overall, the participants in the two groups experienced increased competence, autonomy, and relatedness, and the participants expressed the importance of both being in a natural environment and doing outdoor activities (friluftsliv).

**Conclusions:**

The study makes a valuable contribution to the field of health promotion and rehabilitation pointing to nature and friluftsliv as important elements that offer great potential to community-based health promotion.

## Introduction

Recent studies have shown, that nature is associated to health and mental well-being among the general population (Thompson Coon et al., 2011). This review has shown some promising effects on self-reported mental wellbeing immediately following exercise in nature which are not seen following the same exercise indoors. Engagement with nature has been found to be associated with better mood leading to increased levels of vitality (Joye et al., 2013; Richardson, Cormack, McRobert, & Underhill, Richardson, et al., 2016). The association is especially documented among people with stress related problems (Pálsdóttir et al., 2014) and among people with depression (Korpela et al., 2016). The Attention Restoration Theory offers a possible explanation pointing to a less stressful kind of attention when being in the outdoors and thereby, to natures restorative and stress reducing qualities (Kaplan, 1995). Studies comparing natural and built environments have further shown positive effects on health for people being active in natural environments (Araújo et al., 2019; Lafortezza et al., 2009; Maller et al., 2006; Pasanen et al., 2014; Thompson Coon et al., 2011). In a review, Barnes et al documented the positive mental health benefits from nature experiences including a perspective to different elements of nature and types of experiences in natural areas (Barnes et al., 2019).

Besides being in nature, activities performed in nature are associated with health and well-being (Mygind et al., 2019; Orr et al., 2016; Pretty, 2004). This points to a focus on not only *“being in nature”* or *“doing in nature”* but on the unique combination of *´*being and doing in nature´ A number of studies have e.g., shown, that walking and hiking in nature have positive effects on well-being (Berman et al., 2012; Korpela et al., 2016; Mygind et al., 2019) and on cognitive functioning (Bratman et al., 2015).

Activities in nature are successfully used as a therapeutic tool in relation to adventure and wilderness therapy e.g., intending to address the deleterious effects of urbanization and technification in adolescents (Durr, 2009; Gabrielsen et al., 2019; Gabrielsen & Harper, 2018). The Nordic pedagogic literature points to the Nordic tradition of simple life in nature, *friluftsliv*, as having the potential to positively impact well-being (Andkjær, 2012; Gurholt Pedersen & Haukeland, 2019; Tordsson, 2014). The tradition of *friluftsliv* is understood as a non-formal culture in the Nordic countries that within the past 30 years has been transformed and used as a pedagogical and therapeutic tool intending to improve e.g., environmental awareness, personal, and social competences, as well as health and well-being (Andkjær, 2012). *Friluftsliv* includes non-competitive activities in natural settings and organized in small groups. The pedagogic approach is inspired by experiential learning, emphasizing active participation, responsibility, and ownership from the participants. A special feature in *friluftsliv* is the overall focus on nature and place, and on the process, which leaves time for reflection and discussion and to focus on individual needs (Andkjær, 2012; Gurholt Pedersen & Haukeland, 2019; Tordsson, 2014). Simple life and activities in nature, *friluftsliv*, understood as a Nordic tradition and concept of being and doing in nature, may be an important asset in national as well as local health promotion or rehabilitation strategies.

Non-communicable diseases such as cancer, heart disease and diabetes are responsible for 73% of all global mortality (GBD 2017DALYs and HALE Collaborators, 2018), and one in three adults live with more than one chronic condition (Marengoni et al., 2011). Also, the prevalence of mental health problems is increasing globally (Whiteford et al., 2013). In Denmark, the proportion of people experiencing poor health has increased, and especially mental health is regarded as a major public health challenge (Danish Health Authority, 2018). In Denmark, the municipalities have the primary responsibility for carrying out preventive and health promotion activities, and to undertake the majority of rehabilitation with an ambition to implement interdisciplinary activities in their health and rehabilitation programs based on best practice and current knowledge (The Danish Ministy of Health, 2011).

There is rich literature on the potentials of implementing various types of health promotion programs in community settings at large (Kegler et al., 2011; Weiss et al., 2016). Community-based health promotion programs are important strategies in improving health and well-being of the population and among people living with chronic diseases Thus, there is an increasing recognition of possibilities and benefits of nature-based interventions to improve individuals’ mental health (Bragg & Atkins, 2016; Corazon et al., 2019; Mygind et al., 2019; Shanahan et al., 2019). Outdoor exercises and adventure therapy has been implemented in rehabilitation programs indicating that short singular exposures to nature and physical activity can potentially improve mental health in the general population (Barton et al., 2012; Durr, 2009; Mygind et al., 2019). Only few studies, however, have examined the potential benefits of *friluftsliv* or activities in the naturel environment in clinical populations e.g., people with mental problems and/or chronic diseases showing overall positive effects but also mixed effects on included outcomes of psychosocial wellbeing (Mygind et al., 2019). Studies on the relation between nature, *friluftsliv* and health generally face methodological challenges due to the complexity of pathways between immersive *friluftsliv* and mental health outcomes as *friluftsliv* combines characteristics of nature, activities and experiences (Thompson Coon et al., 2011). Furthermore, previous studies varies greatly in settings, context, time frames, recruitment and characteristics of the participants (Mygind et al., 2019). Consequently, research on the potential benefits for mental health and well-being of community-based outdoor health promotion and rehabilitation programs is sparse.

In the current study, we seek to obtain a better understanding of this by asking the following research questions:

- How do people with mental or chronic physical health problems experience a local municipal outdoor health promotion or rehabilitation program?
- How may these programs contribute to the participants’ health and well-being?

## Methods

The evaluation was designed as a qualitative multiple-case study based on data from the *Healthy in Nature* project using both qualitative focus group interviews and observations. The design allowed us to gain detailed and nuanced knowledge of the chosen cases by pointing to 1) how the participants have experienced the programs, 2) what elements in the programs have been important, 3) possible differences between the two groups of participants.

### *The* Healthy in Nature *project*

The *Healthy in Nature* project was carried out by the Danish Outdoor Council from 2017 to 2020 and financed by the National Lottery Funds and involving ten municipalities in Denmark. The aim was to implement *friluftsliv* into local outdoor health promotion and rehabilitation programs (OHPR programs) in each participating municipality, resulting in a total of 27 local programs (Wengel et al., 2020). The participating municipalities and partner organizations included in the project (e.g., local outdoor associations/clubs) had some flexibility in defining and organizing the activities for each OHPR program. This enabled the activities to be adapted to and embedded in the local settings. Activities, e.g., hikes and games in the outdoors, were led by instructors with a speciality in rehabilitation (e.g., physiotherapist) and/or the use of nature and outdoor education (e.g., a nature guide or outdoor instructor).

Program theories or logic models are widely acknowledged among health promotion professionals as important planning and evaluation tools (Chen et al., 1999; McLaughlin & Jordan, 1999), and the implementation of the local programs was guided by an overall program theory developed by the National Institute of Public Health, University of Southern Denmark, based on current literature and knowledge in the field (Jakobsen et al., 2018). For both groups, the model included the following two core elements: first, group-based programs, carried out in an outdoor natural setting; second, a focus on health promotion, rooted in a community setting, adjusted to the target group, and engaged with partners from the civil society. A key element in the program was the integration of *friluftsliv*, which covers a broad spectrum and mix of various elements ranging from physical activities (e.g., play, hiking, mountain biking, and training) to more sensory activities (e.g., mindfulness, forest bathing, fishing, and gathering around a bonfire). The group-based health promoting activities not only involved participants as a part of a group, but also allowed the participants to alternate between active participation and withdrawal. The overall assumption was that the outdoor activities improved health and well-being by combining the natural setting with the individual experiences and social interactions. The programs targeting people with chronic physical health problems predominantly included a focus on physical activity aiming to increase recovery and the ability to cope with the disease. The programs targeting people with mental health problems included a focus on sensory aspects aiming to decrease symptoms of stress, depression, and anxiety.

### Design

The study is qualitative aiming at identifying experiences from the participant groups and interpret and understand these in relation to the chosen theoretical framework. The underpinning methodological framework is inspired by philosophical hermeneutics (Denzin & Lincoln, 2000; Gadamer, 2008) and the design is a multiple-case study study (Flyvbjerg, 2001; Merriam, 1998; Stake, 2005), which serves to provide more extensive descriptions and understandings of the OHPR program.

Five cases (out of 27 possible) were selected: two cases with a focus on adults with chronic physical health problems such as diabetes, heart disease, cancer and obesity (participant group 1); and three cases with a focus on adults with mental health problems such as stress, anxiety and/or depression (participant group 2). Participants were included in the local community health promoting or rehabilitation programs by either: a) referral from their general practitioner (GP); b) self-referral; or c) referral from the public employment centre (people on sick leave or unemployment benefits experiencing mental health problems). Cases were selected based on the criteria of geographic variation and programs running during the data collection period.

The sample included five focus groups of three to six participants giving a total of 14 women and seven men. The participants were recruited by the instructors and selected by purposive sampling based on the following criteria: 1) the participants were willing and comfortable to participate in the interview, 2) both men and women were represented if possible, 3) participants with both a high and a low participation rate were included (see Table I for an oversight of the five OHPR programs included in the study and the participants in the focus group interviews). Qualitative focus group interviews (Rabiee, 2004) were used in order to capture the group dynamics of the participant groups and thereby identify nuances and different experiences from the cases selected. All focus group interviews were conducted on sight in a natural setting used in the programs (see Table I), with the same researcher (TTKW) as moderator. Additionally, a series of participant observations and interviews with local project managers were completed and used as supplementary data to support findings from the primary interview data with program participants.

**Table I.** Description of the participants in the five groups included in the qualitative multiple-case study evaluating the Danish Healthy in Nature project

Municipality	Duration of the course (No. of weeks; hours pr. week)	Activities	Setting	N (course)	N (focus group)
Participant group 1: Adults with chronic physical health problems					
Fredensborg	10; 2	Physical activities (walking, fishing, orienteering, cooking at the fireplace, basic skills for outdoor activities, foraging, and learning about nature).	Forest with access to facilities such as shelter, fireplace and toilet.	16	2 women 3 men
Vordingborg	12;2	Physical activities (walking and mountain biking, cooking at the fireplace, foraging, and learning about nature).	Different places in a natural setting.	12	4 women
Participant group 2: Adults with mental health problems					
København^a^	9; 3	Therapy sessions around the bonfire, meditation in a natural setting, walks, learning about nature, light physical activities.	Forest/park with access to facilities such as shelter, fireplace and toilet.	12	3 men
Nordfyn	25; 1.5	Meditation in a natural setting (forest bathing, walks, learning about nature, games and light physical activities).	Forest/park with access to facilities such as shelter, fireplace and toilet.	8	4 women
Vejle	4;2	Mindfulness and nature-based therapy (walking in silence, sitting around the fireplace, learning about nature and sensory activities).	Forest.	9	4 women 1 man

^a^The course was for men only, which is why only men participated in the interview.

### Data collection and analysis

Data was collected in the period 4 June to 20 June 2019. By using focus group interviews, it was possible to collect information on the participants’ life situation, opinions, attitudes and experiences with the OHPR programs (Brinkmann & Tanggaard, 2015). The focus group interviews were carried out using a semi-structured interview guide including questions about the perception of a) valuable elements of the programs e.g., the specific activities carried out, the contextual setting, the instructor, the organization and the social network b) the general experience of participating in the program and c) experiences of changes in health behaviour or state of health. The questions were created by the researchers, with reference to the research question, and informed by literature and the theoretical framework. Focus group interviews were conducted onsite in nature during the OHPR program and held immediately after the last activity sessions of the courses. The focus group interviews were conducted by one of the authors and lasted between 45–75 min. All focus group interviews were audio recorded and transcribed verbatim by the interviewer, including field notations of non-verbal expressions.

Data were analysed based on the theoretical framework (Self-Determination Theory) following an abductive analytical approach (Charmaz, 2006; Timmermans & Tavory, 2012). In this way the analysis was guided by the theoretical concepts, but also permitted us to be open to consider a variety of interpretations of the data before arriving at our findings. The analysis was guided by Braun et al.’s 6-phase guide to qualitative reflexive thematic analysis (Braun et al., 2016) using Nvivo11 for systematic coding. The analysis allowed an in-depth and nuanced understanding of the participants’ experiences of the program, and how it may have contributed to their health and well-being. The findings were discussed among the authors and presented to keypersons in the participating municipals for validation.

### Theoretical framework

Improved well-being and quality of life was considered a main outcome of the OHPR programs. Self-Determination Theory (SDT) offers a useful theoretical framework in this study, as it focusses on how self-determination, being in control of one’s own life, is essential for health and well-being (Ryan & Deci, 2000). The theory proposes that humans have three innate psychological needs that are the basis for intrinsic motivation: *autonomy, competence*, and *relatedness*. Autonomy is the perception of being in charge of one’s own behaviour and initiative. Competence refers to the feeling of being competent and capable to progress and develop using acquired skills. Relatedness is a feeling of belonging and being part of a social environment in order to thrive. Meeting these three basic needs helps to motivate the initiation and long-term maintenance of health-promoting behaviours (Ryan et al., 2008; Silva et al., 2014), and, as such, SDT served as a relevant framework for understanding the OHPR programs based on the individuals’ experiences.

### Methodological considerations

The study offers a deep insight and knowledge about a limited number of programs and about the participants’ experiences from participating in the programs. In line with its qualitative design, the study did not seek absolute truths, but attempted to reveal the essence of a phenomenon. As Flyvbjerg (1993, 2001) argues, the qualitative case study is a good methodological way to gain insight in everyday life phenomena, and further offers the possibility to discuss generalization on the background of case studies (Flyvbjerg, 1993, 2001). This type of research is valuable to the field of health promotion or rehabilitation because it makes a valuable contribution to knowledge production and to future programs.

Doing qualitative research generally face challenges related to the role of the researchers needing to be aware of and critical reflect on their own preconceptions and conjectures (Denzin & Lincoln, 2000; Kvale & Brinkman, 2015). In the design and process we tried to cover for this by 1) being a group of researchers discussing results critically; 2) validating the results by presenting and discussing preliminary results to both selected groups of participants and to the projects groups; 3) discuss the results drawing on literature and other relevant studies.

Our measure for well-being was the participants´ own experiences from participation in the programs. The study did not involve any objective measure for the effect and for the change in their well-being. In line with this, the selection of respondents to interviews may be critical, and often there is a tendency for the most positive respondents to sign up or be chosen to participate. These possible problems were covered for by applying a critical perspective to the results, being four researchers and using different methods as well as by involving other studies and theory in the discussion. The overall assessment is that the respondents have answered honestly to our questions in the interviews and that the results present valuable knowledge on benefits of the programs studied to the two target groups. It will be possible to argue for a pragmatic generalization to other groups and other programs.

The abductive nature of the thematic analysis means that the analysis was guided by, but not restricted to, the chosen theoretical framework. Other theoretical perspectives could have been applied and examined in the study, e.g., Banduras concept of self-efficacy (Bandura, 1977, 1990), which could have resulted in a more behaviour-altering and change-related perspective. The ambition, however, was to gain a deeper understanding of the health-related effects of the project and Self-Determination Theory enabled a closer exploration and understanding of the participants´ experiences, motivations, and self-perceived well-being.

The participants were interviewed by the end of the project and the data collected told us how the project is experienced immediately after the project. The design of the study allowed us to build conclusions about the importance and effects of the project in the short term, but it was not possible to conclude any long-term effects. To have a lasting effect the projects needed to be anchored in the way that the participants would continue to be active in nature doing *friluftsliv* and continue to experience the positive effects on their well-being.

### Ethics

The participants were introduced to the study both verbally and in writing, and they all gave informed consent to participate in the study. Prior to each interview, the participants were informed about their right to call off the interview at any time. They were promised confidentiality and they are anonymized in the text. The Danish Data Protection Agency approved the study in accordance with the Act on Processing of Personal Data (University of Southern Denmark, Journal no. 10.567). The study was conducted in accordance with the Declaration of Helsinki. The researchers had no interests of conflict.

## Results

The results of the thematic analysis based on SDT identify the following themes: 1) autonomy; 2) competence; 3) relatedness, and 4) being and doing in nature. The programs overall increased the participants’ experiences of autonomy, competence, and relatedness and thereby their self-perceived health and well-being. The results clearly show that the participants experienced that the OHPR programs had a positive impact on their health and well-being in different ways. The participants expressed that being in the outdoors and doing activities together was important for their benefit from the programs. In relation to the character of the activities, some differences in the experiences were identified between the two participant groups.

### Autonomy

The results showed that many participants from both participant groups experienced a high degree of autonomy as a consequence of participating in the programs. Their experience of autonomy increased particularly because they experienced nature and the outdoors as an open space with more flexibility and room for individual needs and choices.

I like the open air and the space out here. We are all bringing something, yet different, and we are free to decide for ourselves how much we will share one day and another. I think it works in a very respectful and natural way. You can say that the relationship between us is as natural as being in nature. (Participant, group 2)

The participants appreciated being in a group doing activities in the outdoors (*friluftsliv*) together and still having the opportunity to withdraw without leaving the group. They were able to choose their own level of participation, which was especially important on days where they did not feel the energy or motivation to participate.

And you can step back a bit, and there are some others who contribute and share, while you can be by yourself. I think it means a lot that you don´t have to participate. It gives peace of mind that focus is not on you all the time. (Participant, group 1).

In addition, the experience of being and doing in the outdoors was important to the participants as this removed the focus on illness. The welcoming context and the fact that they were not constantly being asked about their health was of great importance to the participants as they could be themselves and not only be `the sick person´. This seems especially important to participants with mental health problems.

It’s the time and quietness. We aren’t put under pressure. You don´t have to perform, you aren´t at the doctors and need to be finished in ten minutes. We have time to sit and listen to each other. (Participant, group 2)

These experiences made the participants feel good and more motivated to go outdoors and be active, not because others told them to do this, but because they were driven by an internal motivation. The interviews performed with the project managers supported these findings as they experienced being in the outdoors was less demanding, allowing the participants to stay in the background if they needed to.

### Competence

Overall, the participants expressed that they developed new competencies through the OHPR programs. For both groups, these competencies involved experiencing new ways of using the outdoors, which could be applied to their daily life. Many participants in both participant groups experienced that they gained knowledge about nature and became more familiar with being in the outdoors during the activities. To see and recognize small things, e.g., various edible plants, had a positive impact on their motivation to be in the outdoors.

Just to open your eyes and see those small things in nature. After all, it´s those things that give the extra energy you need. (Participant, group 1)

Several participants from both groups emphasized that the increased knowledge and experiences of nature and *friluftsliv* encouraged them to be more active in the outdoors. They expressed that it inspired and enabled them to show others how to use nature and *friluftsliv*.

I want to take a break, and I want to take my kids out here showing them the peace of just being here. And also, to show them how to use the outdoors. I used to think that being in the outdoors with the kids, you go and look for skeletons from animals, that’s the way, or you collect spruce cones. But the idea of just lying down and looking up, I´d really like to teach them that!. (Participant, group 2)

The results show minor but significant nuances as the participants with chronic physical health problems (participant group 1) emphasized that being challenged and developing new physical skills enabled them to master new activities (e.g., mountain biking or walking in rough terrain), whereas finding peace in nature was experienced as a new competence among the participants with mental health problems (participant group 2) (see Table II).

**Table II.** New competencies—differences between the two participant groups

Being challenged and developing new physical skills enabled them to master new activities	Finding peace in nature was experienced as a new competence
“I also think my mobility has improved. I now feel more like moving. I’m the kind of person who sits at my desk most of the time, and I experience that I feel much better and now have more stamina.” (Participant, group 1) “And I had never thought I was going to get on a mountain bike, never ever. And I had actually been looking forward to today, right until I realized that there is no foot brake, shit shit shit shit … But I did it and it was definitely a victory. That was really important.” (Participant, group 1)	“Instead of thoughts just running around in my mind, I have become better at observing my thoughts and now better understand why these things happen.” (Participant, group 2) “I went to the tree, then I looked up, and when I looked up at the tree, there were just five different directions of branches. Suddenly like seeing my brain and then it just dripped down on me. And I’ve really thought about that since, the thing about looking up in the trees and finding peace. (…) Finding peace, that (the course) has definitely given me.” (Participant, group 2)

Participants with chronic physical health problems (participant group 1) emphasized that it was rewarding for them to be challenged and to try different activities during the program. They experienced that the small group (between 8–12 persons) was important for their courage to try new activities. This gave them a greater self-confidence and the courage to continue working with day-to-day challenges in the future. Several participants expressed that they felt happy when they gained these feelings of success and overcame personal barriers. The interviews with the project managers supported this result, as they experienced that some of the participants, due to the outdoor activities, crossed personal boundaries leading to greater self-confidence.

Participants with mental health problems (participant group 2) expressed that through the programs they improved their abilities to use nature and *friluftsliv* to find peace. They experienced improved self-reflective skills, which enabled them to explore their own feelings, and they learned how to loosen up the body, which all made everyday life easier. Also, they expressed that it was important to them that the programs took place in the outdoors. There was no time pressure during the outdoor activities, and they did not feel, they had to live up to the norms and guidelines that otherwise are present in society. The flexibility in relation to time and space enabled them to be present and not think about their daily lives, which was experienced as a new competence. The gained tools, such as meditation, helped them to better cope with everyday problems and challenges, and the new competences of experiencing peace in the outdoors could be integrated in their everyday life.

The nuances and differences between the two participant groups might be explained by the triangle of supporting environments (Bengtsson & Grahn, 2014) pointing to four levels of engagement with the physical and social environment depending on the persons subjective experience of well-being. The participants with mental health problems focus on finding peace in nature might be related to the Attention Restoration Theory (ART) (Kaplan & Kaplan, 1989) and psycho-evolutionary theory (Ulrich, 1984, 1991). The participants with chronic physical health problems emphasized trying new activities and being challenged, which can be related to studies on wilderness therapy often used in relation to young people with mental problems (Gabrielsen et al., 2019).

### Relatedness

The participants from both participant groups emphasized that it meant a lot to them to participate in a group-based program. They quickly became part of a group and experienced a high degree of relatedness with the other participants in the group. Overall, the participants felt that building social relationships was crucial to their motivation to attend and participate in the OHPR programs.

The unity that we have, it´s absolutely fantastic. And once again that you´re looking forward to coming here. We´re doing really well in the team. Really, really well. And the energy that we have, and we get from each other, it´s also amazing. And support, it´s the same story. The support we get from each other is also important. (Participant, group 1)

According to the participants, the use of nature and *friluftsliv* in the programs made it easier for them to talk to each other and to take part in conversations, e.g., about the weather or the various plants and animals they saw. Developing confidence and mutual trust in the group was important to all participants as it made it easier for them to open up to each other. Also, the experience of confidence and trust enabled them to challenge themselves e.g., by trying new and challenging activities.

The participants experienced that it was easier to build social relationships and meet more diverse people in OHPR programs compared to indoor programs. In the outdoors, and as a central element in *friluftsliv*, the participants felt more dependent on each other and needed to take responsibility for and help each other, e.g., when they had to cross a small stream on the walk, or when they did lend one another gloves, when it was cold.

Compared to the groups that are inside and those I´ve been part of, being in the outdoors I experience a greater openness and relaxation – that´s, among people. (Participant, group 2)

This finding was confirmed by several project managers who emphasized that the feeling of group dynamics developed in the outdoors and through *friluftsliv*, where participants quickly opened up to each other, addressed each other, and helped each other.

Several participants emphasized, that the bonfire served as an important gathering point for developing social relations. This experience was shared by the project managers. Sitting around the fireplace, the participants felt a greater degree of freedom compared to sitting in an indoor setting, and this experience obviously contributed to and enhanced their conversations and helped building social relationships.

I think the nice thing is that we´re slowly opening up ourselves – more and more. We have this circle sitting around the fire. (Participant, group 2).

There´s definitely something about the bonfire, it gives us some sort of gathering place. (Participant, group 2)

### Being and doing in nature

Overall, the participants appreciated that the activities took place in the outdoors as they here experienced less restricted boundaries and more space for diversity. At the same time, they expressed the importance of the activities and thus emphasized the importance of both *being and doing* in nature.

The participants appreciated activities in the outdoors that stimulated their senses, and made them experience peace, which they often found difficult in their everyday life. Participants with mental health problems (participant group 2) particularly indicated that quiet activities were of great importance to their benefit of the program. Many participants found that the natural setting was ideal for calm and sensory activities, such as meditation and sensory exercises, which stimulated their senses, eg. when they listened to the wind, watched wildlife or tasted edible plants.

When we walked around, it was exactly the point of walking around and feeling with your feet, and it probably could´nt have worked inside. Here we walk around outside on branches and the ground is uneven, “crick crack” when you step on something. I was about to step on a frog that came bouncing, it just would´nt have happened inside. (Participant, group 2)

The project managers confirm this, as they also experienced sensory and meditative activities in the outdoors, suitable for participants activating their senses and stimulating them in different ways. The project managers even experienced a kind of symbolism in the outdoors, e.g., the roots of the trees, symbolizing the importance of having a good foundation in life and being able to stand firm. This allowed for spontaneous analogies making the participants reflect on and discuss their own life situations.

Most of the participants with chronic physical health problems (participant group 1) and some with mental health problems (participant group 2) expressed that it was important to them to participate in fun and exciting exercises, e.g., collaborative exercises or strength training with natural forest elements. It was important to them that the activities could be adapted to their individual skills, and that everyone could participate. Participants with chronic physical health problems (participant group 1) in particular expressed that they preferred fun and playful activities without competition, and they experienced such activities positive for the social relationships. They emphasized that there were many elements in nature and *friluftsliv* that made the activities more playful, e.g., cooperation and the less formal setting. This made them forget the element of exercise, because they enjoyed the activity.

You actually disappear a bit in it, and sometimes it really just feels like you have been out playing instead of exercising. (Participant, group 2)

I think it´s nice. I think it´s nice that we can say, now we´re throwing all those stiff flippers we have and then we tumble. (Participant, group 1)

Overall, integrating nature and *friluftsliv* into health promotion and rehabilitation programs was perceived as having a positive impact on the participants’ experiences of autonomy, competence and relatedness and thus on their well-being.

## Discussion

The aim of this qualitative study was to explore and seek a better understanding of how people with chronic physical or mental health problems experience a local outdoor health promotion or rehabilitation program and to understand how these programs may contribute to health and well-being. Overall, the themes identified in the case study seem to agree with the tenets of the SDT theory. Results showed that the participants experienced that the *Healthy in Nature* project had a positive impact on their health and well-being in different ways. The local programs have increased the participants’ experiences of autonomy, competence, and relatedness and thereby their self-perceived health and well-being. The participants expressed that being in nature and doing activities together was important in order to benefit from the programs. Some differences between the experiences of the two participant groups were identified according to the character of the activities.

The main findings in the study underline the positive impact of nature to well-being, which is well-known and documented in a series of other studies (Richardson et al., 2016; Thompson Coon et al., 2011). In line with previous research (Joye et al., 2013), the thematic analysis based on SDT show that the participants experience that the programs with nature and *friluftsliv* have an impact on their health and well-being in different ways. The results confirm findings from previous studies indicating that being in nature has the potential to improve health and well-being for people with stress related problems and chronic diseases (Bowler et al., 2010; Han et al., 2016; Pálsdóttir et al., 2014; Ray & Jakubec, 2014).

### The social perspective

The social perspective—relatedness—seems to be the most prominent aspect of the findings expressed by the participants in both groups. Albeit the elements are interrelated, the participants clearly value and highlight the importance of relatedness. This indicates that relatedness might be of particular importance, and that it can be understood as a basic or overall element that makes it possible to experience competence and autonomy and thereby well-being. Nature and *friluftsliv*, as it is practiced in the programs, seem in different ways to facilitate the participants experience of relatedness. First the participants highlight being in nature as a new setting, where they can meet people in the same situation in a new way. Here they experience norms and rules on how to behave as much more open and flexible compared to traditional indoor facilities. In this setting, they are not met with the same expectations, and focus is not on diagnosis, treatment, and illness, but rather on being and doing together. The results highlight the qualities in the natural setting as more flexible and open, with no or only few pre-set norms and with a greater acceptance of individual needs and choices. This feature can be understood in the light of the concept of affordance as it was originally formulated by Gibson (Gibson, 1977) and can even be related to a phenomenological understanding of nature’s *open communication* with no pedagogic intensions (Tordsson, 2002).

### Friluftsliv*—doing in nature*

In correspondence to previous findings, the study indicates that it is not a matter of just being in a natural setting but rather a combination of both being and doing in the outdoors that is important to the participants´ perceived benefits of the programs (Orr et al., 2016; Pretty, 2004). This points to activities and to pedagogics, and the Nordic tradition of *friluftsliv* with its focus on the process (non-competition), small groups and involvement. These key-elements seem to make an ideal framework according to the aim of working with challenged groups, motivation, and well-being. This way of being and doing in nature seems to balance the flexibility of the process and the aim of achieving results in the programs. It contrasts other studies introducing and working with wilderness therapy, which rather focus on wilderness and challenging activities besides aiming at other target groups and using other methods (Gabrielsen et al., 2019; Gabrielsen & Harper, 2018).

With a focus on the natural setting and on activities, *friluftsliv* seem to support the participants perceived feeling of not being seen as a patient but as a person. According to the participants, this is much easier in the outdoors, where the space is experienced in a different way and feels more open, and where the focusing is on doing activities together in a small group.

Building and gathering around a bonfire is important to the participants as it seems to facilitate their experiences of both autonomy, competence, and relatedness. Many of the participants mention the bonfire, and it might be seen as an icon or symbol of being and doing in nature as part of the concept of *friluftsliv* in the traditional Nordic outdoor culture (Andkjær, 2012). The participants need to work together to build and start a fire, which takes some effort and depends on a certain level of competence to light the fire and get it going. The fire is a central place where the group can be together and discuss their experiences. At the same time, the fireplace is a flexible place where participants can withdraw and return freely if they feel like, which serves to facilitate their feelings of autonomy.

### Target groups and implementation

The findings indicate that the use of nature and *friluftsliv* has an impact on both participant groups’ experiences of autonomy, competences, and relatedness and thus their feeling of well-being. However, minor differences were identified between the two participant groups included in the study, and the findings point to the importance of designing a program with a close reference to the specific participant group. The group of adults with chronic physical health problems such as diabetes, heart disease, cancer and obesity (participant group 1) experience that physical activity, exercise and new challenging activities are important to them and that these qualities motivate them and make them want to join the program (see Table II). The other group, adults with mental health problems such as stress, anxiety and/or depression (participant group 2), seem to be more attracted to calm and sensory activities as they experience these activities important to their feeling of well-being (see Table II). This is in line with earlier studies examining interventions and effects to people with stress related problems and people with depression (Korpela et al., 2016; Pálsdóttir et al., 2014). It is possible to argue that being and doing (*friluftsliv*) in nature is a good way to work with health promotion and rehabilitation, but also that the design of the program needs to focus on the specific target group. This could suggest a possible involvement of the target group in the design of the program and the choice of activities, which is part of the pedagogical thinking in *friluftsliv* (Andkjær, 2012; Tordsson, 2014).

The output of the OHPR programs is related to the implementation delivery setting and the external contexts such as the environment. The programs are operating in a complex system and the public health problems targeted by the programs are considered complex, which points to a need for more detailed scientific knowledge on different interventions, groups and in different contexts (Barnes et al., 2019; Thompson Coon et al., 2011). Although, the high level of tailoring and the complexity of the programs challenge the generalizability, the bottom-up approach has several advantages such as increasing sustainability. The approach taken in this case study is to understand how these programs may impact health and well-being of specific groups of people. Further research could benefit from focusing on implementation of the programs and the local capacity building i.e., the possible impact of the implementation and delivery strategies. In addition, further research should/needs to include larger experiments based on quantitative methods and involving control groups to build upon qualitative insights and provide generalizable knowledge.

## Conclusion

The evaluation of the *Healthy in Nature* project gives a better understanding of how people with mental or chronic physical health problems experience a local municipal OHPR program and a better understanding of how the programs contribute to health and well-being. Minor differences between the two groups of participants were identified indicating that OHPR programs need to be adapted the target groups of interest when designing the program.

Overall, the Outdoor Health Promotion and Rehabilitation programs were successful in promoting self-perceived health through increased competence, autonomy, and relatedness to adults with mental or chronic physical health problems. Moving away from a purely health professional approach and including other elements embedded in being and doing in nature seems to be a good way to address people in local health promotion and rehabilitation programs.

## Implications

The study gives reason to recommend that municipalities should continue to develop and implement health promotion and rehabilitation programs using nature and *friluftsliv* to impact health and well-being of different groups of citizens with health-related problems. It can be argued that this type of research is valuable to the field of health promotion or rehabilitation and makes a valuable contribution to knowledge production and to future programs.

Further research, however, is needed on the effect of different methods and programs to different participant groups in different contexts. Future research should address the actual implementation of the OHPR programs and examine its effectiveness, expanding the collaboration between research and practice in municipalities. Suggestions could be implementing local case studies with specific target groups focusing on different program elements possibly integrating citizen involvement. Ways of structuring organization or networks aiming at anchoring the effects of a municipal program need to be investigated further. Consideration should be given to initiating larger experiments involving control groups including quantitative methods to build upon qualitative insights.
